# Treatment of missing data in Bayesian network structure learning: an application to linked biomedical and social survey data

**DOI:** 10.1186/s12874-022-01781-9

**Published:** 2022-12-19

**Authors:** Xuejia Ke, Katherine Keenan, V. Anne Smith

**Affiliations:** 1 School of Biology, Sir Harold Mitchell Building, Greenside Place, KY16 9TH St Andrews, UK; 2 School of Geography and Sustainable Development, Irvine Building, North Street, KY16 8AL St Andrews, UK

**Keywords:** Simulation study, Missing data, Bayesian networks, Social science, Multimorbidity

## Abstract

**Background:**

Availability of linked biomedical and social science data has risen dramatically in past decades, facilitating holistic and systems-based analyses. Among these, Bayesian networks have great potential to tackle complex interdisciplinary problems, because they can easily model inter-relations between variables. They work by encoding conditional independence relationships discovered via advanced inference algorithms. One challenge is dealing with missing data, ubiquitous in survey or biomedical datasets. Missing data is rarely addressed in an advanced way in Bayesian networks; the most common approach is to discard all samples containing missing measurements. This can lead to biased estimates. Here, we examine how Bayesian network structure learning can incorporate missing data.

**Methods:**

We use a simulation approach to compare a commonly used method in frequentist statistics, multiple imputation by chained equations (MICE), with one specific for Bayesian network learning, structural expectation-maximization (SEM). We simulate multiple incomplete categorical (discrete) data sets with different missingness mechanisms, variable numbers, data amount, and missingness proportions. We evaluate performance of MICE and SEM in capturing network structure. We then apply SEM combined with community analysis to a real-world dataset of linked biomedical and social data to investigate associations between socio-demographic factors and multiple chronic conditions in the US elderly population.

**Results:**

We find that applying either method (MICE or SEM) provides better structure recovery than doing nothing, and SEM in general outperforms MICE. This finding is robust across missingness mechanisms, variable numbers, data amount and missingness proportions. We also find that imputed data from SEM is more accurate than from MICE. Our real-world application recovers known inter-relationships among socio-demographic factors and common multimorbidities. This network analysis also highlights potential areas of investigation, such as links between cancer and cognitive impairment and disconnect between self-assessed memory decline and standard cognitive impairment measurement.

**Conclusion:**

Our simulation results suggest taking advantage of the additional information provided by network structure during SEM improves the performance of Bayesian networks; this might be especially useful for social science and other interdisciplinary analyses. Our case study show that comorbidities of different diseases interact with each other and are closely associated with socio-demographic factors.

**Supplementary Information:**

The online version contains supplementary material available at 10.1186/s12874-022-01781-9.

## Background

Bayesian networks (BNs), first proposed by Pearl [1], are a flexible statistical tool for encoding probabilistic relationships with directed acyclic graphs (DAGs) [2]. BNs have a wide range of applications, including developing expert systems for predicting diseases [3], disclosing diffusion of messages in social networks [4], reconstructing gene regulatory networks [5], and inferring neuronal networks [6] and ecological networks [7]. However, BNs are still only rarely applied to population health and social science questions. Relatedly, use of survey data for BN structure learning is limited.

**Fig. 1:**
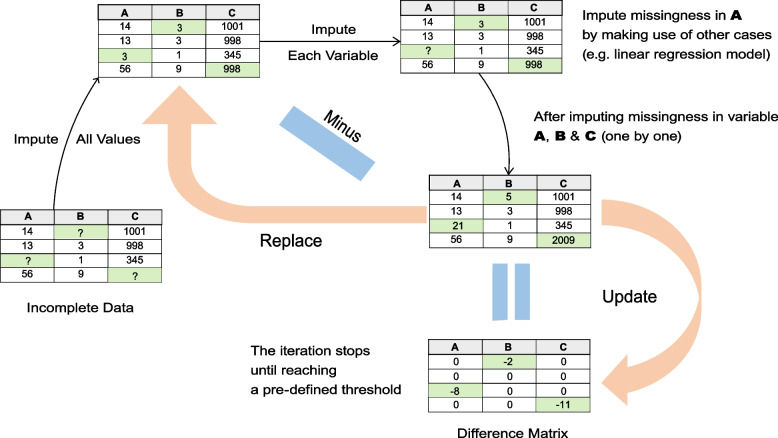
Schematic diagram of Multiple Imputation by Chained Equations approach. For a given incomplete dataset, MICE firstly imputes all missing values via univariate imputation methods. Then it removes the imputed values from variables one by one and creates a model by using the other complete samples. After that, it imputes missingness in each variable in turn using the created model and the remaining variables. These steps are repeated until the data is completed. It then subtracts this new completed data from the initial imputed values to get a difference matrix. The new completed data then becomes the starting point for the next iteration. The whole process is iterated until a pre-defined threshold on the difference between initial imputed and new completed data is met

**Fig. 2:**
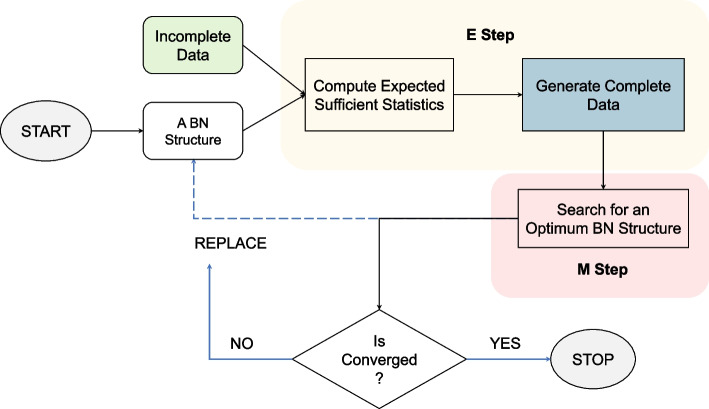
Schematic diagram of Structural Expectation-Maximization algorithm. SEM has two components: E-step and M-step. It considers a BN structure for the incomplete data at the very beginning. Then it applies the iterative two steps, alternating E-step and M-step. E-step estimates the values of missing data by computing the expected statistics using the current network structure. The M-step maximizes the scoring function and updates the resulting network structure. These two steps are repeated until convergence is met

Compared with other fields of study, for instance, experimental biological systems, missing data are more pervasive in observational and survey data. There are plentiful causes, including item missingness, e.g., unanswered questions in questionnaires, data entry errors, or subject missingness, e.g., patients dropping out in longitudinal research, or missing samples. Missing data not only reduce overall statistical power and precision, but can lead to biased inferences in subsequent data analysis [8]. Taking a popular method of listwise deletion (e.g., undertaking analysis only on those complete cases without any missing data) as an example, its statistical power and precision would be inevitably reduced because of the decreased sample size.

Based on the different processes leading to the missingness, every missing data pattern can be generally classified into three categories - missing completely at random (MCAR), missing at random (MAR), and missing not at random (MNAR) [9]. This nomenclature is widely used in statistical data analysis and is also referred to as the missing data mechanisms. MCAR occurs if the missingness is unrelated to both unobserved and observed variables. Data are said to be MAR if the missingness is related to observed variables but not to any unobserved variables given the observed ones. MNAR is the most complicated because its missingness relates to both unobserved and observed variables [9]. These three patterns cause different levels of risks of bias in data analysis. For instance, listwise deletion analysis in MAR and MNAR data would yield more biased estimates than MCAR [10].

Multiple imputation by chained equations (MICE) is a popular multiple imputation method used in biomedical, epidemiological and social science fields. It is designed to impute missing data values under the missing data assumption MAR [11, 12]. Compared to single imputation, multiple imputation methods are less biased because they take account of the uncertainty of the missing data by combining multiple predictions for each missing value. MICE uses a divide and conquer approach to replace missing values for all variables in the data set: it focuses on one variable at a time and makes use of other variables to predict the missing values in that focused variable. Figure 1 illustrates how MICE imputes missing values for a given incomplete data set. Firstly, it imputes all values by using univariate imputation methods (e.g., replace missing values by the median of a single variable) to create a starting point. Then it removes the imputed values from each variable in turn and creates a model (e.g., a linear regression model) using the complete samples. This model may or may not include all variables in the dataset. After that, it imputes the values in each variable using this model and other values in the remaining variables. These steps are repeated until the data is completed. Then it subtracts this completed data from the starting point to get a difference matrix. To make this difference close to 0, the whole process is iterated, using the just completed data as a new starting point, until a pre-defined threshold on the difference between the starting point and new completed data is met. Depending on the features of the focused variable, MICE employs different multivariate regression models to predict the missing values (e.g., logistic regression for binary dependant variables). In epidemiology and clinical research, multiple imputation can enhance reliability of inferences based on data with values missing at random (MAR); however, the same procedures are not suitable for MNAR data, and thus further work is required to address MNAR data in a multiple imputation framework [8].

Learning BN structure from incomplete data is quite challenging. Depending on the missing data mechanisms (e.g., MNAR or MAR), learning would be biased if we simply delete incomplete observations. However, while BNs can theoretically consider completion of the dataset, to do so for all missing values in all possible configurations would increase computational time infeasibly (exponential increase per missing data point) [13].

The structural expectation-maximization (SEM) algorithm makes BN structure learning from incomplete data computationally feasible by changing its search space to be over structures rather than parameters and structures. SEM iteratively completes the data, then applies the standard structure learning procedures to the completed data [13]. Similar to the standard EM algorithm [14], SEM involves two steps - expectation (E-step) and maximization (M-step). Figure 2 shows the basic principle of SEM algorithm. Firstly, it considers a BN structure (e.g., an empty one) for the incomplete data. Then it applies the iterative two-step, alternating E-step and M-step. The E-step estimates the values of missing data by computing the expected statistics using the current network structure. The M-step maximizes the scoring function and updates the resulting network structure. This continues until convergence is met [15]. The framework of SEM was first proposed by Friedman [16]. His simulation results suggest that although there is a degradation of learning performance with an increased percentage of missing data, SEM shows promise for handling data involving missing values and hidden variables [16]. Friedman [15] later improved his work so that SEM is not limited to using scoring matrices like minimal description length (MDL) or Bayesian Information Criterion (BIC) that only compute the approximations to Bayesian posterior probability, enabling direct optimizations of the Bayesian posterior probability that incorporates prior information (e.g., Dirichlet priors) over network parameters into the learning procedures.

In this study, we evaluate methods for addressing incomplete data using a simulation framework. Simulation provides a vital mechanism for understanding and evaluating the performance of approaches before applying them to real-world cases. Here we simulate multiple incomplete categorical data sets, including three different missing data mechanisms, various number of variables and amounts of missing data. We concentrate here on categorical, or discrete, data due to its ubiquity in population health and social science data (e.g., categorical survey responses, presence or absence of disease). We then evaluate and compare the performance of MICE and SEM with each other and with the standard expedient of using only samples without missing data, by comparing their resulting network structures with the original network structure.

We then apply the best working method (SEM, see Results) to a real-world health and social survey dataset to investigate concurrent chronic diseases in the US elderly population. Multimorbidity (the concurrence of two or more chronic diseases in an individual) places an enormous burden on individuals and health systems, and is expected to grow more in importance as populations age [17–19]. Researchers have used a variety of methods to unpick the complexity of combinations of diseases, and identify clusters and risk factors [20, 21]. Among these, BNs have great potential to tackle such complex problems and can help us understand multimorbidity as a complex system of biosocial disadvantage. In our network analysis, we investigate the interactions between presence and treatment of several chronic diseases, cognition, and their associations with health behaviours and other factors including race, gender and socioeconomic status.

## Methods

### Overview of our simulation

**Fig. 3:**
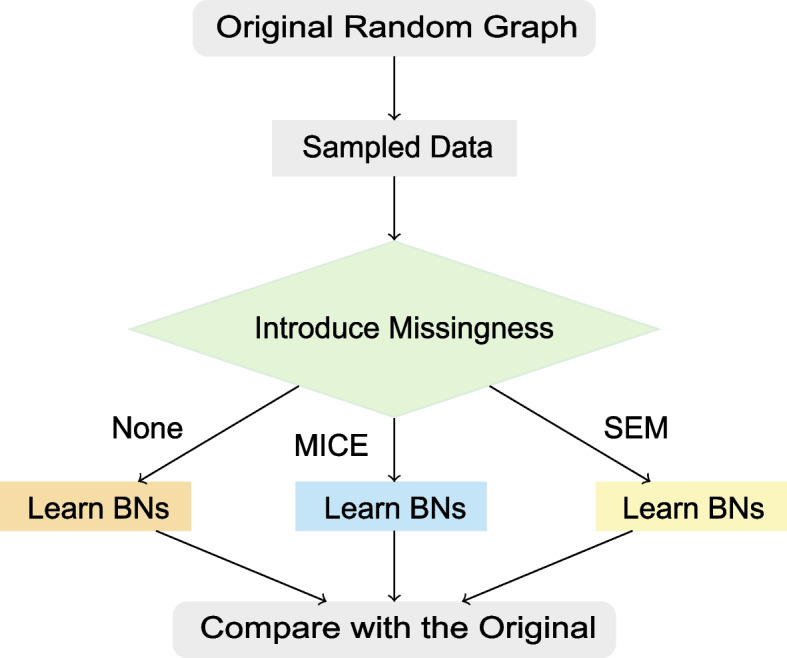
Flowchart of our simulation approach

Figure 3 shows an overview of our simulation approach. We compare the performance of MICE and SEM on incomplete categorical (discrete) data, and both against doing nothing (e.g., using only complete cases). The main steps are as follows:

1. Generate a random graph. This random graph is also referred to as the original structure in the final step for comparison.

2. Sample data points from the random graph to get the complete data.

3. Introduce missing values to the complete data.

4. Learn the Bayesian network structure, either: (a) from all complete cases, (b) from the data set completed via MICE, or (c) using SEM.

5. Compare learned Bayesian network structures with the original structure.

We analysed networks with numbers of variables ranging from 2 to 20. For each number of variables, we analysed a range of missing proportions from 0.1 to 0.6 at intervals of 0.1. Each variable number/missing proportion was repeated 100 times. We completed the whole analysis for each of 1000, 5000 and 10,000 sampled data points.

### Simulated data

#### Random networks and sampled data

We first generated a randomly connected network structure with the specified number of nodes (variables) using method Ide’s and Cozman’s Generating Multi-connected DAGs (ic-dag) algorithm in the function random.graph from R package bnlearn [22]. We set maximum in-degree for any node at 3, and each node had 3 discrete levels. Various descriptive statistics of these random network structures are shown in Additional file 1; the networks had expected changes: increasing out-degrees, reduced density and clustering, and increased diameter with larger networks. We obtained conditional probability tables (CPTs) for each node by generating random vectors from the Dirichlet distribution using function rdirichlet from R package MCMpack [23]. The parameter $\alpha$ of Dirichlet distribution was 0.5 for nodes with parents and 5 for nodes without parents. This provided our random parameterised BN. We then randomly sampled 1000, 5000 or 10,000 data points from the parameterised BN to get our sampled data using the function rbn from R package bnlearn [22].

#### Missing data

For each missing data mechanism, we introduced different amounts of missing data to the sampled data using the function ampute from R package mice [24]. This function requires a complete data set and specified missing patterns (i.e., the variable or variables that are missing in a given sample). We used the default missing pattern matrix for all simulations, in which the number of missing patterns is equal to the number of variables, and one for each variable is missing. We also used the default relative frequency vector for the missing patterns, so that each missing pattern has the same probability to occur. Thus, the probability of being missing is equal across variables. The data is split into subsets, one for each missing pattern. Based on the probabilities of missingness, each case in each subset can be either complete or incomplete. Finally, the subsets are merged to generate the required incomplete data. The allocated probability for each value to be removed in each subset depends on the specified missing proportion and missing data mechanism [25]:

**MCAR** The missingness is generated by chance. Each value in the sampled data has the same probability to be incomplete and such probability is computed once the missing proportion is specified [25].

**MAR** The probability of each value being incomplete is dependent on a weighted sum score calculated from values of other variables. We used the default weights matrix in our simulation, in which all variables except the missing one contribute to the weighted sum score [25].

**MNAR** Simulating MAR and MNAR data share most procedures during amputation. The only difference is that it is the value of the potential missing value that contributes to the probability of its own missingness [25].

### Bayesian network structure learning

During the whole study, we used the same BN structure learning procedures to learn from data either before processing or after. That is, procedures were all the same for methods “None”, “MICE” and “SEM” in Fig. 3: we used a score and search algorithm, using the BDe score [2] and the tabu search algorithm for searching the best network structure [26]. The imaginary sample size used by BDe was set equal to 1 (default value). A test for the impact of scoring function was performed by also assessing structures learned using the BIC and BDs scores for one dataset configuration (MNAR data, 1000 data points, 0.3 missingness; BDs imaginary sample size set to 1 as default; BIC also used default value for penalty coefficient: log(number data points)*0.5). For “None” and “MICE”, we applied the tabu function from R package bnlearn [22]; for SEM the search was incorporated into the iterative steps as described below.

#### No imputation

We used the complete cases of simulated incomplete data for BN structure learning.

#### Structural EM

We applied the SEM algorithm to the incomplete data using the function structural.em from R package bnlearn [22]. We used the default imputation method (“parents”) in the E-step, which imputes missing data values based on their parents in the current network. We applied tabu search and BDe scoring matrix for structure learning and the default method Maximum Likelihood parameter estimation (mle) for parameter learning in the M-step. The maximum number of iterations was 5 as default.

#### Multiple Imputation by Chained Equations

As all the variables in this study were categorical and unordered, we used the polytomous logistic regression model for prediction using the function mice from R package mice [24]. The number of iterations was 5 as default.

**Fig. 4:**
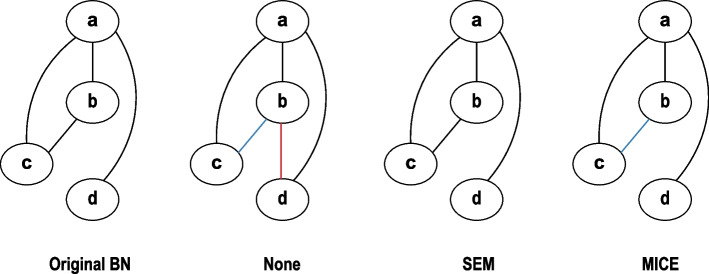
A toy example comparison across four skeleton networks (from left to right): original network, None (complete cases), SEM, and MICE. The original networks work as the reference network for comparison. Blue arcs indicate the arcs that are missed by methods but exist in the original network ($False\;Negative$). Red arcs represent the arcs that are additionally found by methods but not in the original network ($False\;Positive$). Bold arcs are found by methods that are also in the original network ($True\;Positive$)

### Evaluation of recovered network structures

To compare the learned BN structures with the original ones, we compared their skeletons using functions compare and skeleton from R package bnlearn [22]. We compared skeletons, which represent all links in the network as undirected links, to deal with variation of link direction due to different equivalence classes. We explored comparison of equivalence classes, but a single missing/extra link could significantly change equivalence class, giving erroneous results for those dependencies accurately recovered. For example, a link which was directed in the equivalence class of the simulated network could, due to a missing link elsewhere, be undirected in the equivalence class of the recovered network; this would result in not only recording one missing link but also an additional, incorrect, extra link. Comparison of the undirected skeletons resolved this issue. We measured the performance of each method by computing the precision and recall (sensitivity) based on their comparison results. Precision measures the level of a method making mistakes by adding false arcs to the network, while recall evaluates the sensitivity of a method to capturing positive arcs from the targets. Their equations are as follows:

1 $$\begin{aligned} Precision = \frac{True\;Positive}{True\;Positive+False\;Positive} \end{aligned}$$

2 $$\begin{aligned} Recall = \frac{True\;Positive}{True\;Positive + False\;Negative} \end{aligned}$$

where $True\;Positive$ represents finding arcs present in the original structure, $False\;Positive$ represents finding arcs that are not in the original structure, and $False\;Negative$ represents lack of an arc that is present in the original structure (Fig. 4).

We divided the number of variables into 6 groups for analysis: having number of variables 2-5, 6-8, 9-11, 12-14, 15-17 and 18-20. For each group with each missing proportion in each sampled data amount, we performed a one-way ANOVA to test whether there were any statistically significant differences between the means of the three methods. We applied a Bonferroni correction to correct the resulting *p*-values in these multiple comparisons. If there were significant Bonferonni-corrected results (*p* < 0.05) in a variable group/missing proportion combination, we performed the honestly significant difference (Tukey’s HSD) test on the pairwise comparisons between the three methods. For both precision and recall, the same procedures were applied.

### Evaluation of imputed data values

We explored the accuracy of MICE’s and SEM’s imputation, using a subset of the simulations. We extracted the completed datasets from the last iteration of SEM and MICE for each missing mechansim (MCAR, MAR, MNAR) for 1000 data points at missing proportion 0.3, using 10 datasets each of 10 and 20 variables. We calculated the Hamming distance between the imputed datasets from the original (no missing values) simulated dataset. We performed Student’s t-test to test whether there were any statistically significant differences between the means of the Hamming distance of imputed versus original data of the two methods.

### Real-world data application

We use self-reported and nurse-collected data from the United States Health and Retirement Study (HRS) [27–29], a representative study of adults aged 50 and older. We merged the interview data (*N* = 42233) [27] collected in 2016, the harmonised data (*N* = 42233) [29] and the laboratory data (*N* = 7399) [28] that were collected in the same year. As we are focusing on imputation methods, we set any provided imputed values to missing (i.e., to use our method). To ensure a representative sample of older respondents, and due to the focus on multimorbidity, we excluded those aged below 50 (*N* = 279). To ensure biomarker and survey data were collected concurrently, we excluded respondents whose interviews were finished in 2017 and 2018 (*N* = 1394). Our analysis dataset consisted of 29 categorical variables each with two to four levels. Supplementary Table 1 in Additional file 1 shows the detailed description of each variable. This cleaned subset contained 5726 observations, in which only 2688 cases were complete (corresponding to a missingness proportion of 0.53).

We applied the best-working method, SEM (see Results), to this real-world data. Because SEM includes random elements in the algorithm, we averaged across multiple repeats to capture the most complete picture of relationships among real-world variables. To accomplish this, we set different random seeds using the base function set.seed in the R environment, before applying the function structural.em from R package bnlearn [22] (using tabu search and BDe scoring metrics in the M-step, as above). In this way, we learned 100 network structures using the SEM algorithm from the whole incomplete data set. We determined the average network across the 100 repetitions based on an arc strength of each learned structure, calculated from the completed partially directed acyclic graph using the function arc.strength also from bnlearn. As the resulting arc strengths were strongly bimodal (see Results), we included in a final average network all links in the higher mode. While the resulting networks were partially directed, we show as results the skeletons – all links as undirected – because we do not wish to imply causal relationships between these measured variables; we are presenting statistical associations only.

We then further explored relationships among real-world variables based on the network structure by applying hierarchical divisive clustering from the R package igraph [30] to detect the densely connected variables in the learned average network. This identifies community groups consisting of nodes that are densely connected together but sparsely connected to others based on the edge betweenness of the edges without considering the directions.

**Fig. 5:**
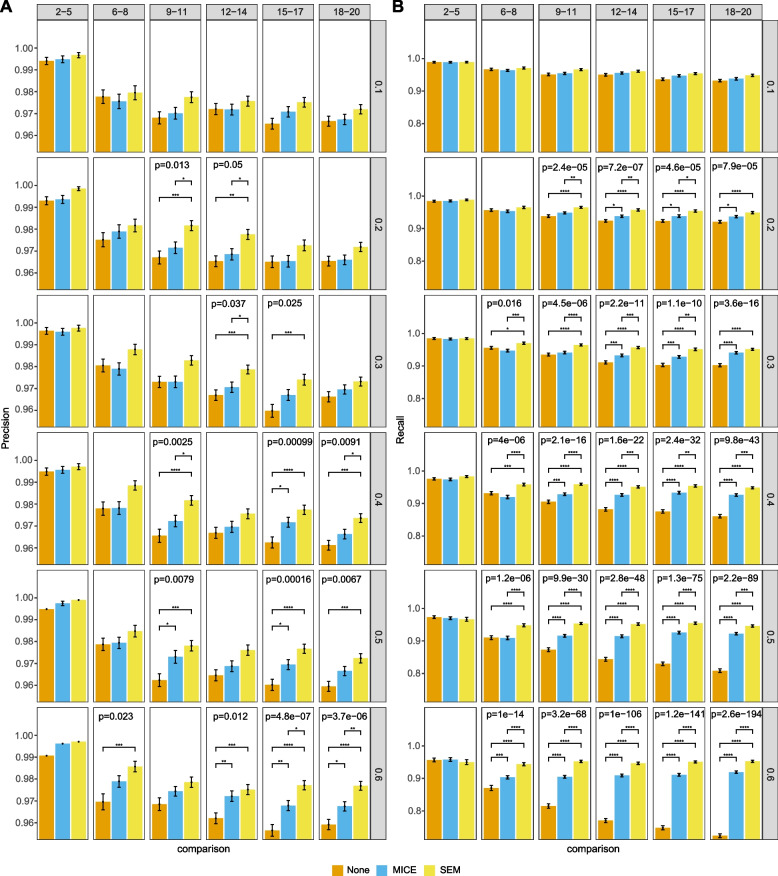
Performance on MCAR data with 1000 data points. Precision (A) and recall (B) of three different methods of handling incomplete data: none, multiple imputation by chained equations (MICE) and structural expectation-maximization (SEM). Rows represent different missing proportions and columns indicate different groups of number of variables. Barplots show means with error bars representing standard error of the mean. Adjusted *p*-values for ANOVAs are displayed in those panels that are significant at least the 0.05 level. Lines representing significant Tukey’s HSD pairwise tests are shown and annotated as: *,* p* < 0.05; **, *p* < 0.01; ***, *p* < 0.001; ****, *p* < 0.0001

**Fig. 6:**
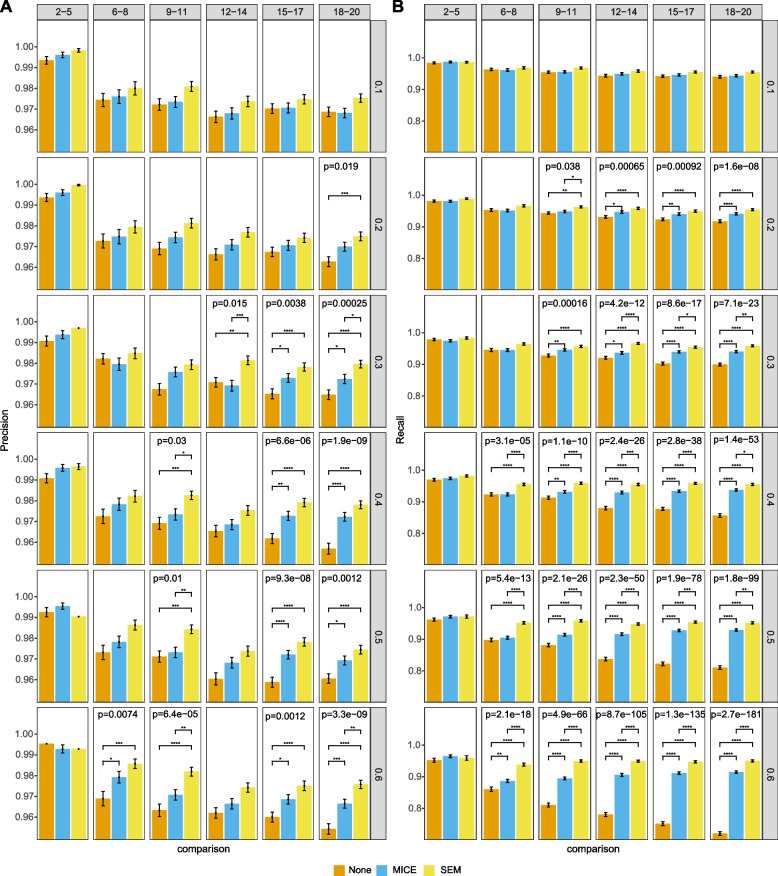
Performance on MAR data with 1000 data points. Precision (A) and recall (B) of three different methods of handling incomplete data: none, multiple imputation by chained equations (MICE) and structural expectation-maximization (SEM). Rows represent different missing proportions and columns indicate different groups of number of variables. Barplots show means with error bars representing standard error of the mean. Adjusted *p*-values for ANOVAs are displayed in those panels that are significant at least the 0.05 level. Lines representing significant Tukey’s HSD pairwise tests are shown and annotated as: *, *p* < 0.05; **, *p* < 0.01; ***, *p* < 0.001; ****, *p* < 0.0001

**Fig. 7:**
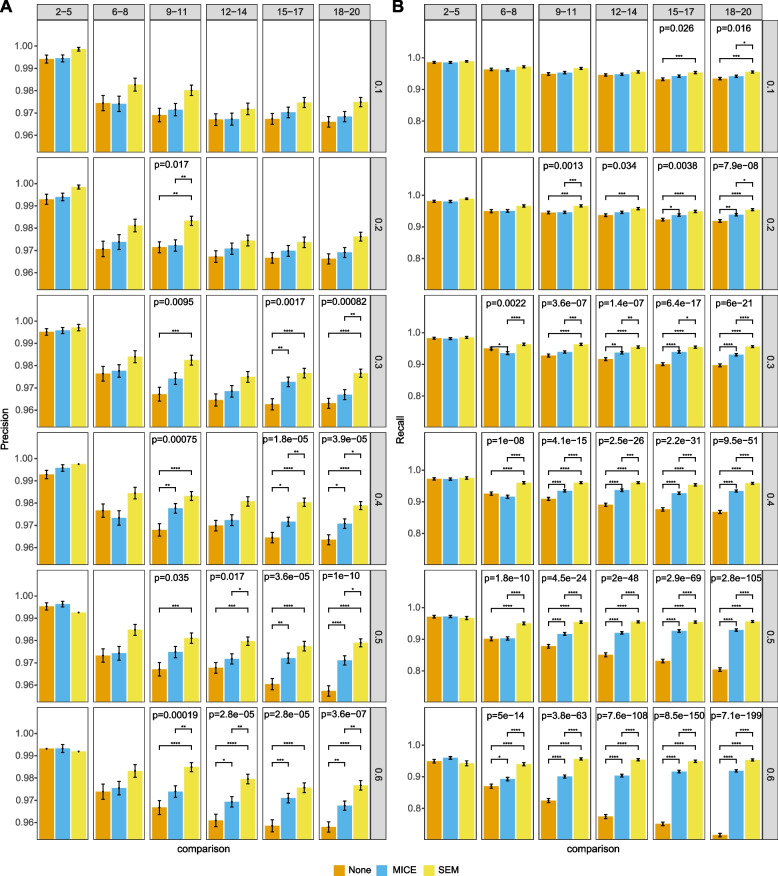
Performance on MNAR data with 1000 data points. Precision (A) and recall (B) of three different methods of handling incomplete data: none, multiple imputation by chained equations (MICE) and structural expectation-maximization (SEM). Rows represent different missing proportions and columns indicate different groups of number of variables. Barplots show means with error bars representing standard error of the mean. Adjusted p-values for ANOVAs are displayed in those panels that are significant at least the 0.05 level. Lines representing significant Tukey’s HSD pairwise tests are shown and annotated as: *, *p* < 0.05; **, *p* < 0.01; ***, *p* < 0.001; ****, *p* < 0.0001

**Fig. 8:**
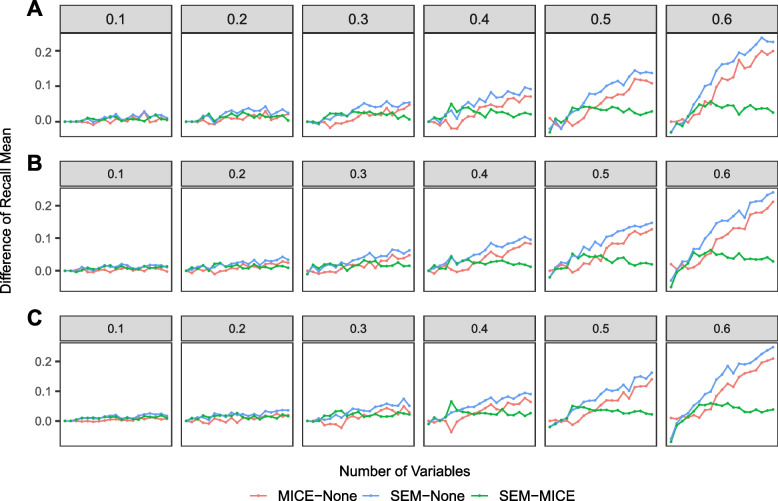
Distribution of the difference in means of recall of three pairwise comparisons among three methods when there are 1000 data points: MICE’s increase over doing nothing (red), SEM’s increase over nothing (blue), and SEM’s increase over MICE (green) A. MCAR data. B. MAR data. C. MNAR data. The y-axis represents the difference of the mean recall (averaged over the 100 simulations). The x-axis represents the number of variables from 2-20. Column panels represent missing proportions

## Results

### Recovered network structures

A total of 1026 scenarios and 102,600 data sets were analysed.

Results of all three missingness mechanisms shared similar features among three levels of sampled data points. Detailed results are shown in Fig. 5 for MCAR, Fig. 6 for MAR, and Fig. 7 for MNAR with 1000 data points. In general, there was enhanced performance of methods of addressing missing data over doing nothing, and better performance of SEM over MICE. There were more significant differences looking at recall than precision. There were more significant differences with increasing proportion of missingness and number of variables. This observation was consistent when there were 5000 and 10,000 data points, although the out-performance of SEM over MICE decreased with 5000 data points and was even less obvious with 10,000 data points. Detailed results for 5000 and 10,000 data points are shown in Additional file 1.

In addition to the pairwise comparisons between the three methods regarding precision and recall, we also compared the performance of each method across the three missing data mechanisms (MCAR, MAR and MNAR) for each level of data points. However, our results did not show any significant differences in performance across the mechanisms.

We summarise patterns of recall across the simulation experiments in Fig. 8 when there are 1000 data points. This demonstrates substantial improvements in performance when using either method (compared to doing nothing), which start to emerge consistently at a 0.3 level of missingness, and increase as levels of missingness and number of variables increases. Generally, SEM outperforms MICE, but the difference does not appear to be conditioned by levels of missingness or missing data mechanism. There is an increase in SEM’s outperformance through low numbers of variables, and then appears to reach an asymptote above 5 or 6 variables. This pattern was also observed when there were 5000 and 10,000 data points (see Additional file 1). However, their scale of observed difference was much smaller than with 1000 data points (differences around 0.01-0.02 compared to 0.1-0.2).

The same general pattern of SEM outperforming MICE, and both imputation methods outperforming doing nothing, also held with the test using the BIC and BDs scores (see Additional file 1).

### Imputed data

**Fig. 9:**
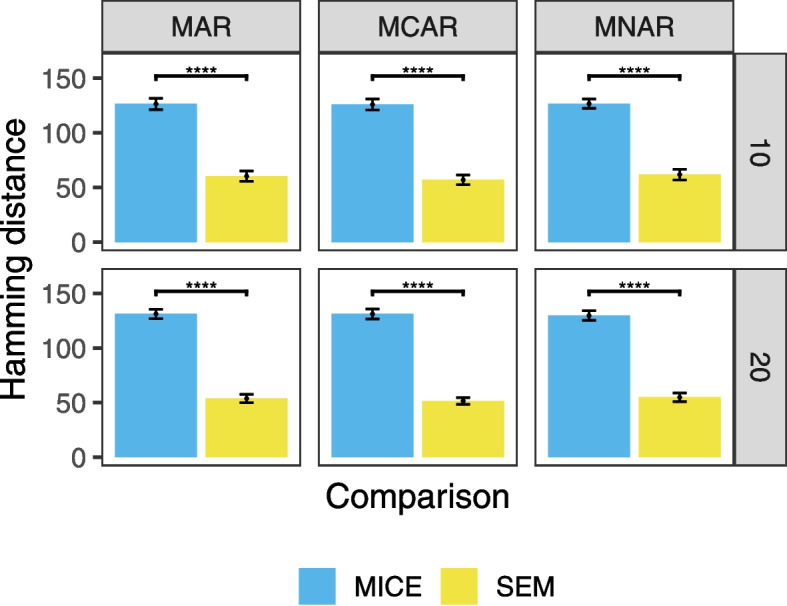
Comparison of the mean Hamming distance of MICE and SEM imputed data from the simulated data at 0.3 level of missingness with 1000 data points, using 10 datasets each of condition. Barplots show means with error bars representing standard error of the mean. Rows represent different numbers of variables and columns indicate different missing mechanisms. Lines representing significant Student’s t-tests are shown and annotated as: ****, *p* < 0.0001

We further compared the performance of MICE and SEM in terms of missing data completion, using 1000 data points with a 0.3 level of missingness. The data completed by SEM in the last iteration is more similar to the original simulated data than MICE (Fig. 9). SEM has a significantly better performance than MICE in data imputation and this finding is consistent when there are 10 variables and 20 variables and across all three missing mechanisms, with *p* < 0.0001 for all comparisons.

### Real-world data application

**Fig. 10:**
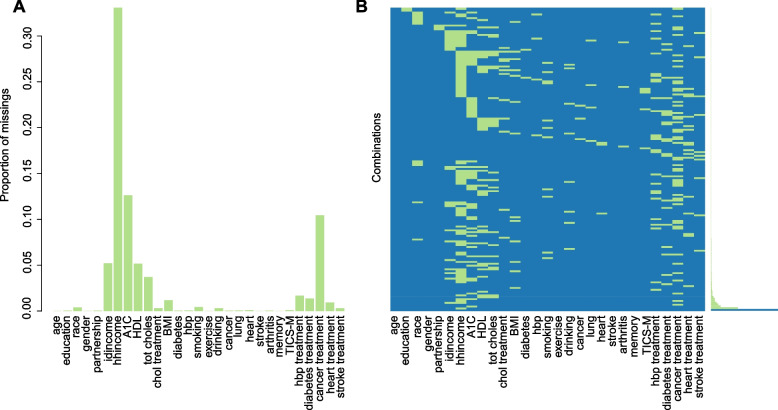
Distribution of missing values in the real-world data set. (A) Proportion of missing values in each variable (named as in Supplementary Table 1 of Additional file 1), shown as a bar chart. (B) Missing patterns, shown as a heatmap with proportions to the right of the plot. Rows represent a single missing pattern (‘Combinations’) and columns variables, with the variable missing in a given pattern coloured green (blue otherwise). The proportion of each missing pattern is shown as a horizontal bar chart to the right of the heatmap (summing to 0.53 for missing patterns). The very bottom row represents the pattern with no missing values, with its proportion bar in blue with value 0.47

Figure 10 displays an overview of the levels of missingness in the cleaned HRS data set. Most have less than 5% of missing values; a few have $\sim$10% or greater, with the highest value being 33.1% missing for household income *hhincome*. There is a large amount of missing patterns that are different combinations of various variables. Only a few variables are missing individually.

**Fig. 11:**
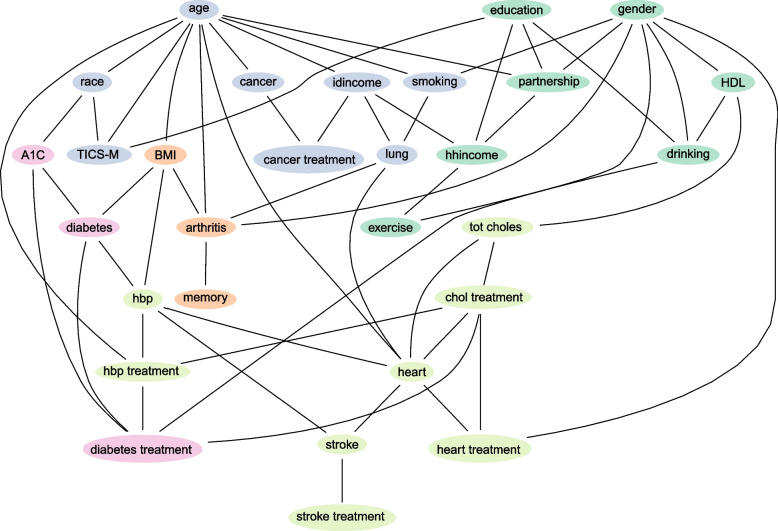
The average network learned from SEM. Nodes are labelled with variable names as found in Supplementary Table 1 of Additional file 1. Nodes are coloured to represent the different groups as discovered by community analysis on the network structure

The arc strengths averaged over the 100 repetitions of SEM applied to this data were strongly bimodal, with individual links having strength 0.87-1.0 (representing presence in 87-100% of the networks) or 0.05 or less. Thus, we generated a final averaged network with arc strengths of 0.87 or greater (Fig. 11).

Five community groups were identified within this network structure (nodes of each community are coloured the same in Fig. 11). Common cardiovascular conditions, such as heart disease, stroke and high blood pressure (HBP), are clustered with total cholesterol level and treatment for those conditions. Diabetes, HbA1c level and diabetes treatment are clustered. Another cluster contains arthritis, self-assessed memory decline and BMI level. Diabetes is directly linked to HBP, HbA1c and BMI levels. The other two clusters contain a mixture of diseases and social factors. Cognitive impairment (TICS-M) is clustered with cancer, lung disease, smoking and race. It is also directly linked to education whereas education clusters with high-density lipoprotein (HDL), drinking, exercise, gender, cohabitation and household income. We find expected links between health behaviours and chronic conditions, e.g., smoking and lung disease. Biomarkers are directly linked to socio-demographic and socio-economic factors, e.g., alcohol use is directly linked to HDL cholesterol level and gender. We also find some unexpected links and clusters: arthritis is directly linked to lung disease, and cancer treatment is directly linked to individual income.

## Discussion

The main aim of this work was to quantitatively evaluate and compare the performance of a common form of imputation (MICE) and SEM on learning BN structures from incomplete data, such as is commonly found in observational health and social datasets. According to our simulation results, as might be expected, both MICE and SEM performed better than no imputation. In addition, significant improvements in recall and precision were observed with SEM versus MICE. This disparity might be explained given that SEM is using additional information, i.e. the structure of the network, to deal with missing data, whereas MICE relies only on the multivariate associations between variables.

We note that SEM performs comparatively well under the MNAR mechanism. This is significant because MNAR is a complex problem to which there is no obvious solution. In MNAR data, a particular value’s missingness rate depends on the real value itself and some unobserved predictors. Although it is theoretically achievable to calculate the missing data rate given the correct set of explanatory factors, in practice it is very hard to find out the combinations of factors that influence the missing rate [31]. Taking an example of blood glucose measurements, people suffering from hyperglycemia will be more likely to drop out of clinical surveys because they feel unwell. However, this assumption is unverifiable using the observed data, and in practice we cannot distinguish between MAR and MNAR data [31]. Multiple imputation methods would therefore generate biased results if we apply them on MNAR data, and the issue can only be addressed by sensitivity analysis to evaluate the difference under different assumptions about the missing data mechanism [31]. In the case of BN structure learning, our results suggest that SEM may be a principled approach to deal with MNAR data. However, this finding should be validated by conducting further experiments under varying MNAR conditions.

The validity of multiple imputation methods also depends on the choices of statistical approaches in analysing the sampled complete data sets and the resulting distribution of estimates for each missing value [8]. More sophisticated approaches are required if the mechanism MNAR appears in different types of variables. Galimard and colleagues [32] recently proposed a new imputation model based on Heckman’s model [33, 34] to address the issue caused by MNAR binary or continuous outcome variables. They then integrated this model into MICE for managing MAR predictors at the same time. We can use function mice.impute.hecknorm from R package miceMNAR [32] to impute incomplete data with MNAR outcome variables and MAR predictors. Although it has been proposed that applying imputation methods on multivariate data before learning BNs can be problematic [32, 35], this novel method might be helpful for the further development of BN structure learning from incomplete data.

While SEM did consistently perform statistically significantly better than MICE, we point out that the differences were relatively small (on the order of <5% for both precision and recall). The overwhelming signal in our results is that imputation is far superior to using only complete cases (e.g., see Fig. 8). SEM can be more computationally intensive than MICE, particularly with higher missing proportion, thus there could be a trade-off between accuracy and computation time. However, these computational times are relatively small (seconds–minutes), thus we still recommend using the better performing SEM.

We showed the usefulness of SEM by applying it to real-world linked biomedical and survey data on chronic diseases, in a dataset which had a high level of missingness. The network we recover from real-world data highlights pivotal interactions among several chronic diseases, health behaviours and social risk factors [20]. As seen in other studies we observe clustering of cardiovascular diseases [36] and metabolic conditions, and treatments for them (e.g. diabetes). Known risk factors of HBP, BMI and smoking either directly or indirectly link to these conditions, although HBP stands apart as being directly linked to diabetes, stroke and heart disease. The connections between cognitive impairment, education and race have been previously observed in the US context [37]. Our analysis also highlights potential areas of investigation. Cognitive impairment is closely associated with cancer, but stands alone from self-assessed memory decline. Cancer treatment is directly linked to individual income, suggesting socioeconomic disparities in cancer treatment, and/or differential survival patterns by income.

Our simulation study showed better performance of SEM, and our real-world case study was able to reveal features of interest from a dataset with high levels of missingness. As in most simulation studies, the main drawback in our simulation is that simulated data sampled from random network is not guaranteed to reflect real data. Our simulation data has two main limitations. First, our simulation used all categorical variables and an even distribution of missing values among variables, which is not very plausible in real-world social science data. For example, some survey questions (e.g., income) will suffer higher levels of missingness due to refusal than other less sensitive ones (e.g., gender). These features probably help to reduce the difference between missing data mechanisms, especially the difference in data with MNAR. This perhaps could also help to explain why there were no significant differences across three missing data mechanisms in our simulation results, particularly with MICE method. Thus, future extensions of this work should incorporate more realistic simulations of mixtures of variable types and uneven missingness patterns. Second, our simulation study deals with cross-sectional, non hierarchical data, and in real social science data observations are often clustered or contain repeat measures from individuals. This can lead to a different, complex and important form of missingness – survey attrition. In future work, we could investigate the application of SEM using more complicated real-world data, using more complex missing patterns (e.g., longitudinal data).

## Conclusion

Our simulation results indicate that both SEM and MICE improve the completeness of BN structures learned from partially observed data. In most circumstances, especially when there are relatively high number of variables and missing values, SEM performs better than MICE. This suggests that making use of extra information from the BN structure within SEM iterations could enhance its capability of capturing the real network structure from incomplete data. In our real-world data application, SEM identified expected interactions between common chronic diseases, and provided additional insights about the links between socio-demographic, socio-economic factors and chronic conditions. Our study suggests that BN researchers working with incomplete biomedical and social survey data should use SEM to deal with missing data.

## Supplementary Information

**Additional file 1:** Supplementary Figs. 1-8, showing simulation results for 5000 and 10,000 data points, Supplementary Fig. 9, showing simulation results of scoring functions BIC and BDs on MNAR data with 1000 data points and 0.3 missing proportion, Supplementary Table 1, showing description of variables in the real-world dataset, Supplementary Tables 2 and 3, showing descriptive statistics of random network structures.

## Data Availability

The data that support the findings of this study are publicly available from the University of Michigan Health and Retirement Study (HRS; https://hrsdata.isr.umich.edu/), based on relevant data sharing policy.

## Abbreviations

MICE: Multiple imputation by chained equations
SEM: Structural expectation-maximization
BNs: Bayesian networks
DAGs: Directed acyclic graphs
MCAR: Missing completely at random
MAR: Missing at random
MNAR: Missing not at random
CPTs: Conditional probability tables
MDL: Minimal description length
BDe: Bayesian Dirichlet equivalent
BIC: Bayesian Information Criterion
BDs: Bayesian Dirichet sparse
HRS: Health and Retirement Study
TICS-M: Telephone interview for cognitive status measurement
HDL: High-density lipoprotein
BMI: Body mass index
HBP: High blood pressure
