# Supplementary material for: Treatment of missing data in Bayesian network structure learning: an application to linked biomedical and social survey data
Source: BMC Med Res Methodol. 2022 Dec 19;22:326. doi: 10.1186/s12874-022-01781-9 (PMC9761946; doi:10.1186/s12874-022-01781-9)
Supplement: Supplementary file 1 — Additional file 1: Supplementary Figs. 1-8, showing simulation results for 5000 and 10,000 data points, Supplementary Fig. 9, showing simulation results of scoring functions BIC and BDs on MNAR data with 1000 data points and 0.3 missing proportion, Supplementary Table 1, showing description of variables in the real-world dataset, Supplementary Tables 2 and 3, showing descriptive statistics of random network structures. [file 12874_2022_1781_MOESM1_ESM.pdf]

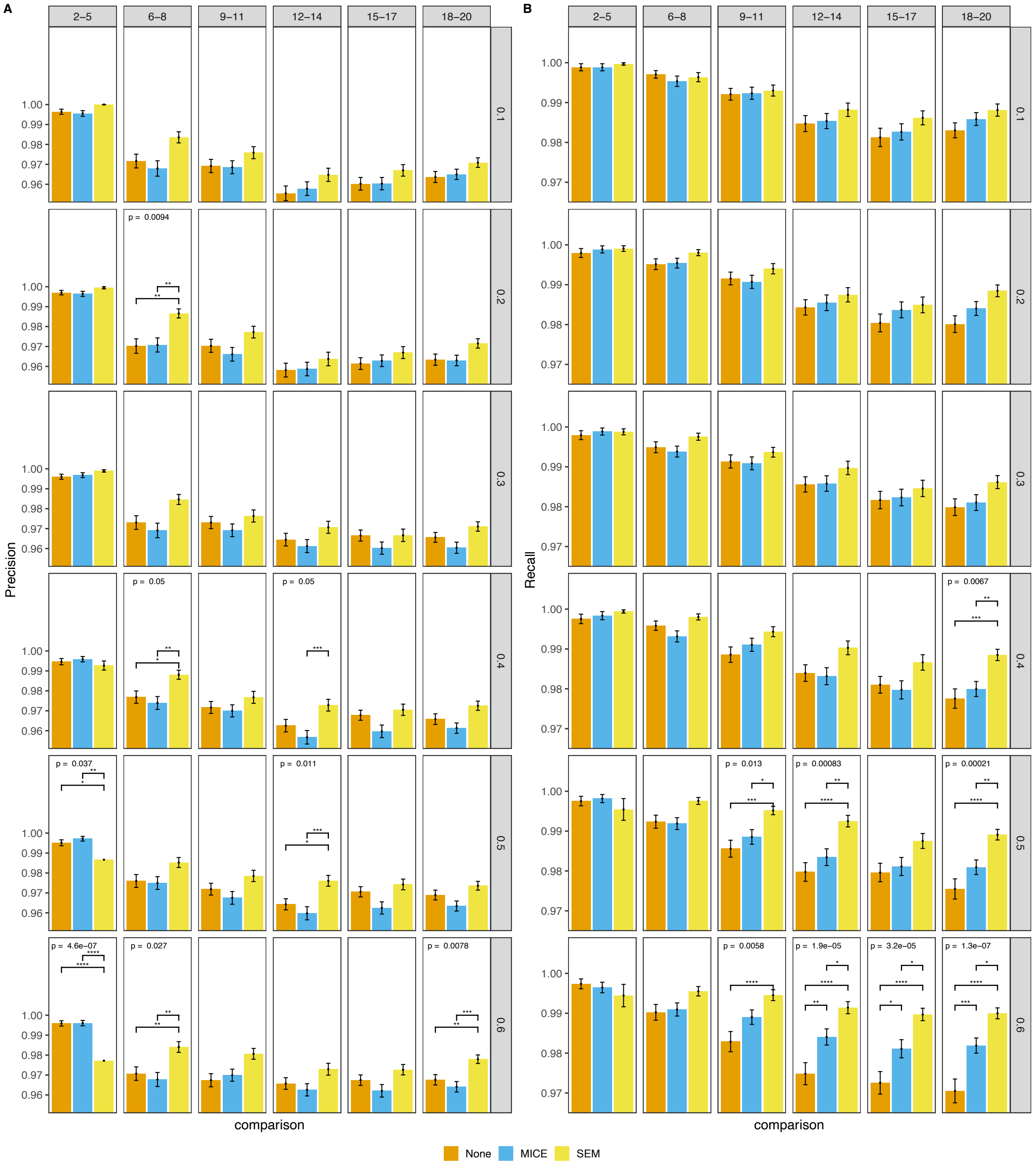

Supplementary Figure 1. Performance on MCAR data with 5000 data points. Precision (A) and recall (B) of three different methods of handling incomplete data: none, multiple imputation by chained equations (MICE) and structural expectation-maximization (SEM). Rows represent different missing proportions and columns indicate different groups of number of variables. Barplots show means with error bars representing standard error of the mean. Adjusted p-values for ANOVAs are displayed in those panels that are significant at least the 0.05 level. Lines representing significant Tukey's HSD pairwise tests are shown and annotated as: *, $p < 0.05$; **, $p < 0.01$; ***, $p < 0.001$; ****, $p < 0.0001$.

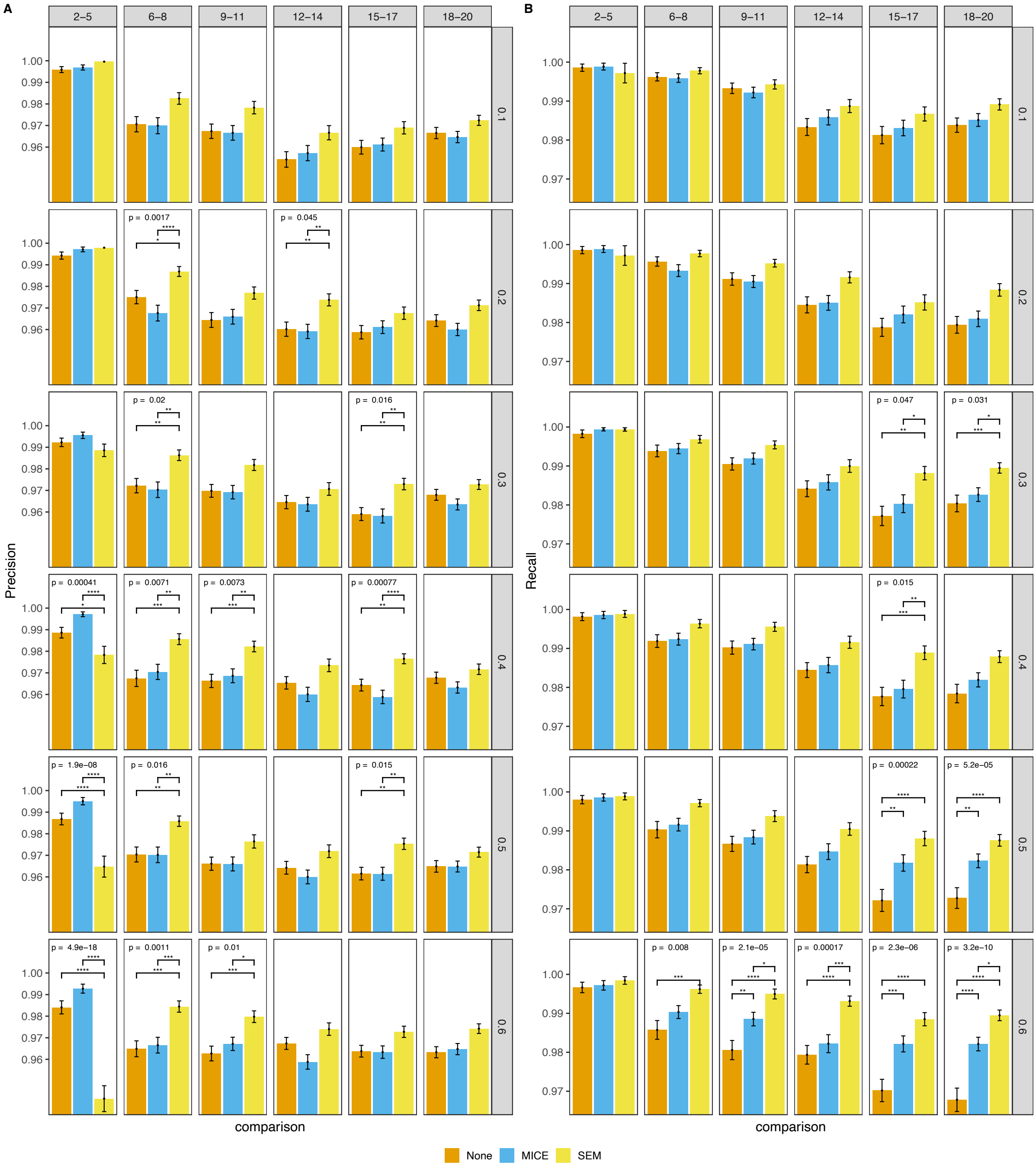

Supplementary Figure 2. Performance on MAR data with 5000 data points. Precision (A) and recall (B) of three different methods of handling incomplete data: none, multiple imputation by chained equations (MICE) and structural expectation-maximization (SEM). Rows represent different missing proportions and columns indicate different groups of number of variables. Barplots show means with error bars representing standard error of the mean. Adjusted p-values for ANOVAs are displayed in those panels that are significant at least the 0.05 level. Lines representing significant Tukey's HSD pairwise tests are shown and annotated as: *, $p < 0.05$; **, $p < 0.01$; ***, $p < 0.001$; ****, $p < 0.0001$.

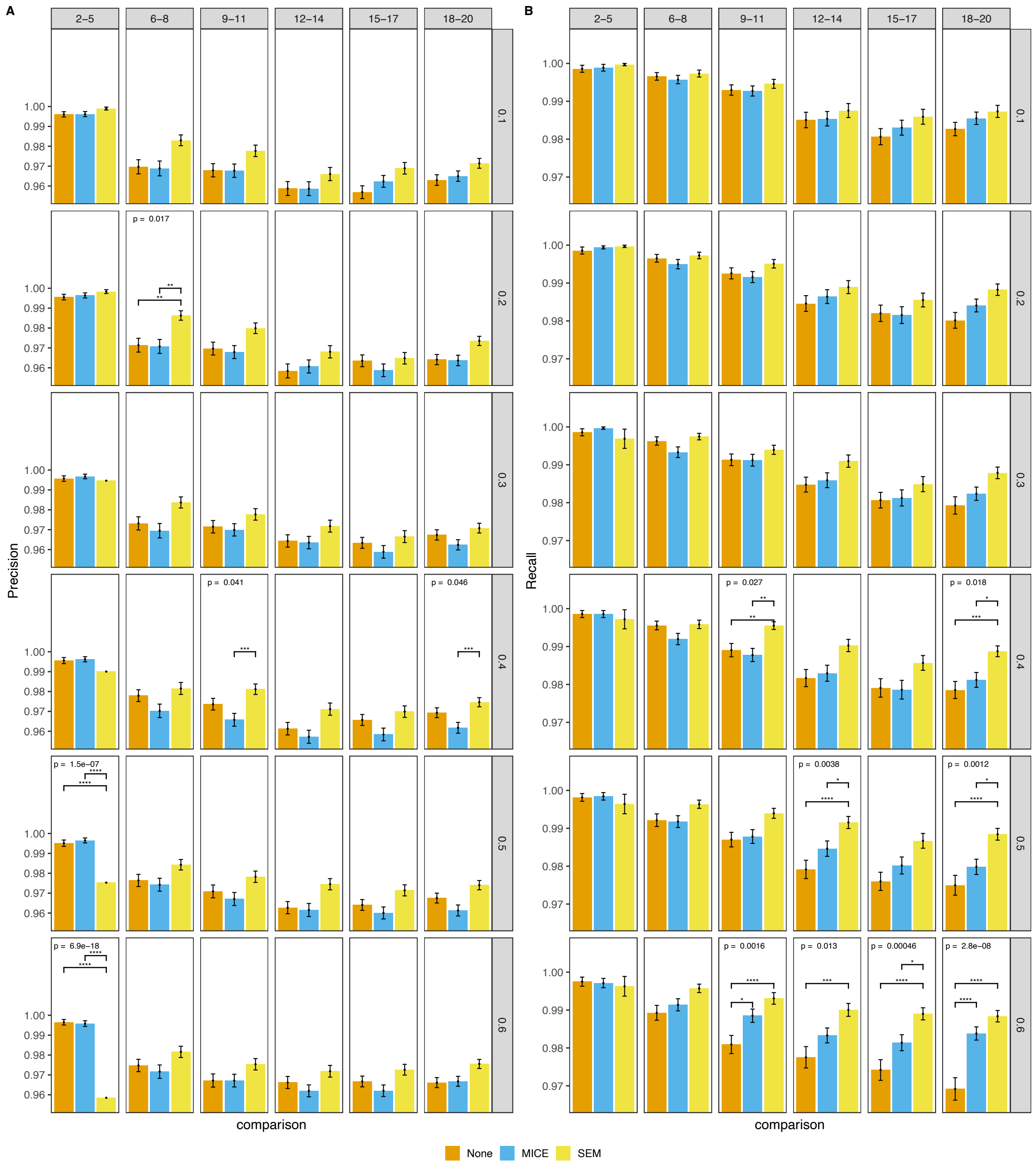

Supplementary Figure 3. Performance on MNAR data with 5000 data points. Precision (A) and recall (B) of three different methods of handling incomplete data: none, multiple imputation by chained equations (MICE) and structural expectation-maximization (SEM). Rows represent different missing proportions and columns indicate different groups of number of variables. Barplots show means with error bars representing standard error of the mean. Adjusted p-values for ANOVAs are displayed in those panels that are significant at least the 0.05 level. Lines representing significant Tukey's HSD pairwise tests are shown and annotated as: *, $p < 0.05$; **, $p < 0.01$; ***, $p < 0.001$; ****, $p < 0.0001$.

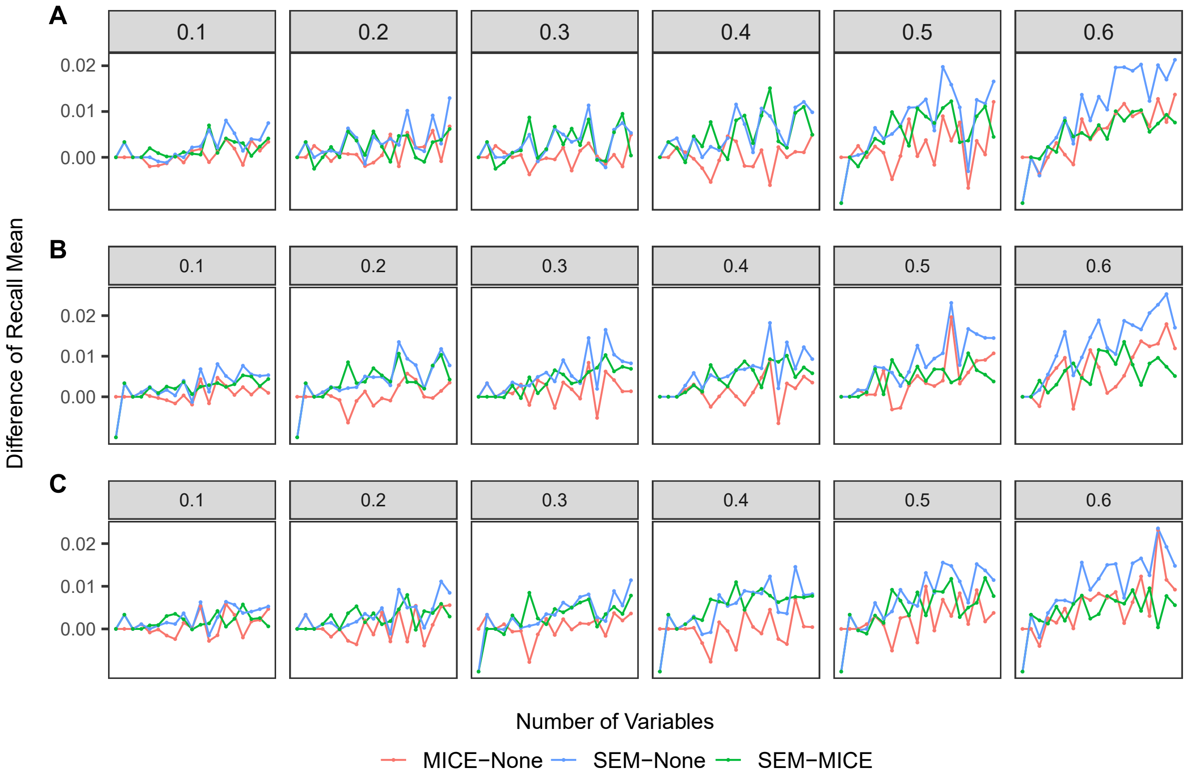

Supplementary Figure 4. Distribution of the difference in means of recall of three pairwise comparisons among three methods when there are 5000 data points: MICE's increase over using only complete cases (red), SEM's increase over nothing (blue), and SEM's increase over MICE (green) A. MCAR data. B. MAR data. C. MNAR data. The y-axis represents the difference of the mean recall (averaged over the 100 simulations). The x-axis represents the number of variables from 2-20. Column panels represent missing proportions.

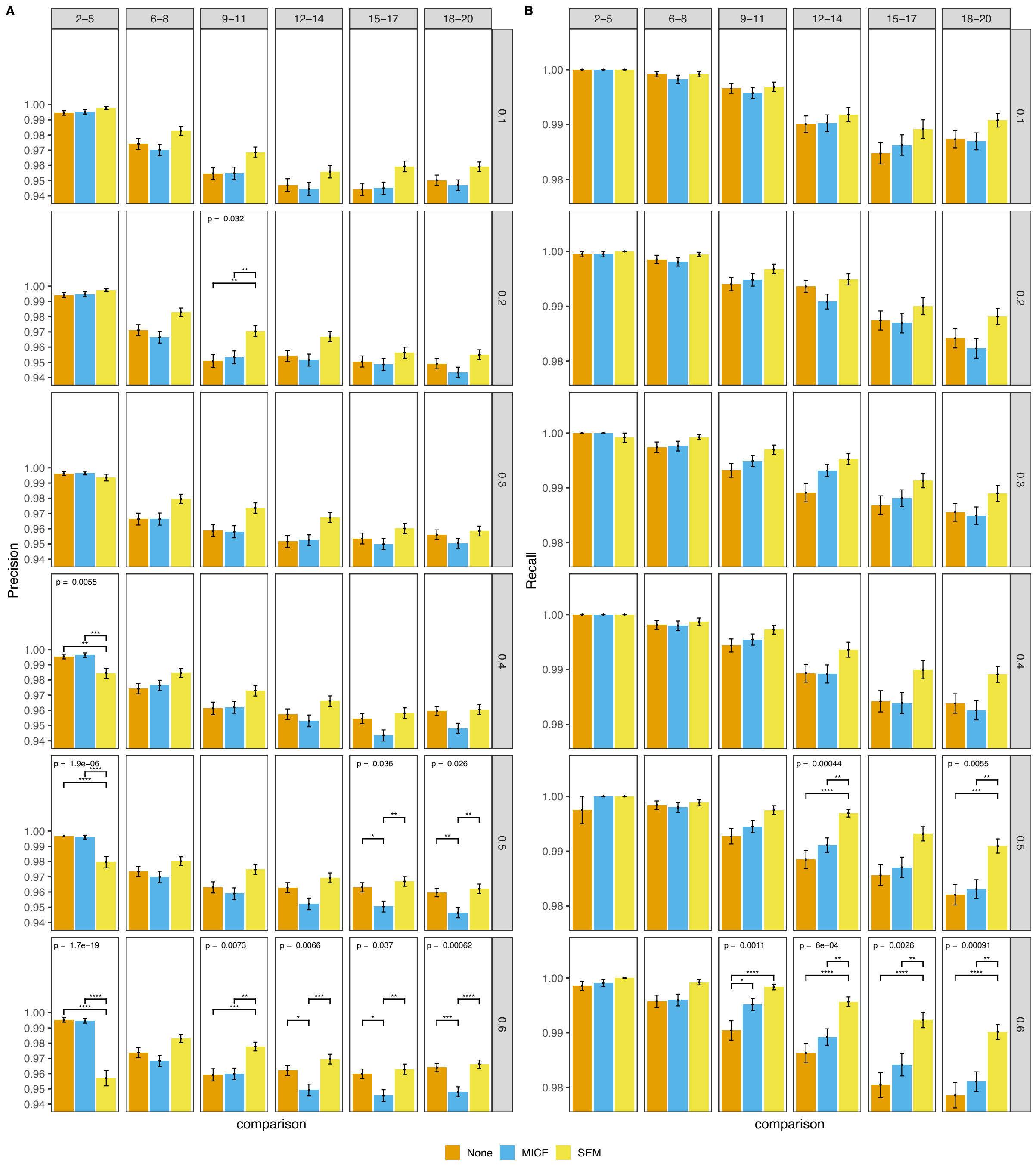

Supplementary Figure 5. Performance on MCAR data with 10000 data points. Precision (A) and recall (B) of three different methods of handling incomplete data: none, multiple imputation by chained equations (MICE) and structural expectation-maximization (SEM). Rows represent different missing proportions and columns indicate different groups of number of variables. Barplots show means with error bars representing standard error of the mean. Adjusted p-values for ANOVAs are displayed in those panels that are significant at least the 0.05 level. Lines representing significant Tukey's HSD pairwise tests are shown and annotated as: *, $p < 0.05$; **, $p < 0.01$; ***, $p < 0.001$; ****, $p < 0.0001$.

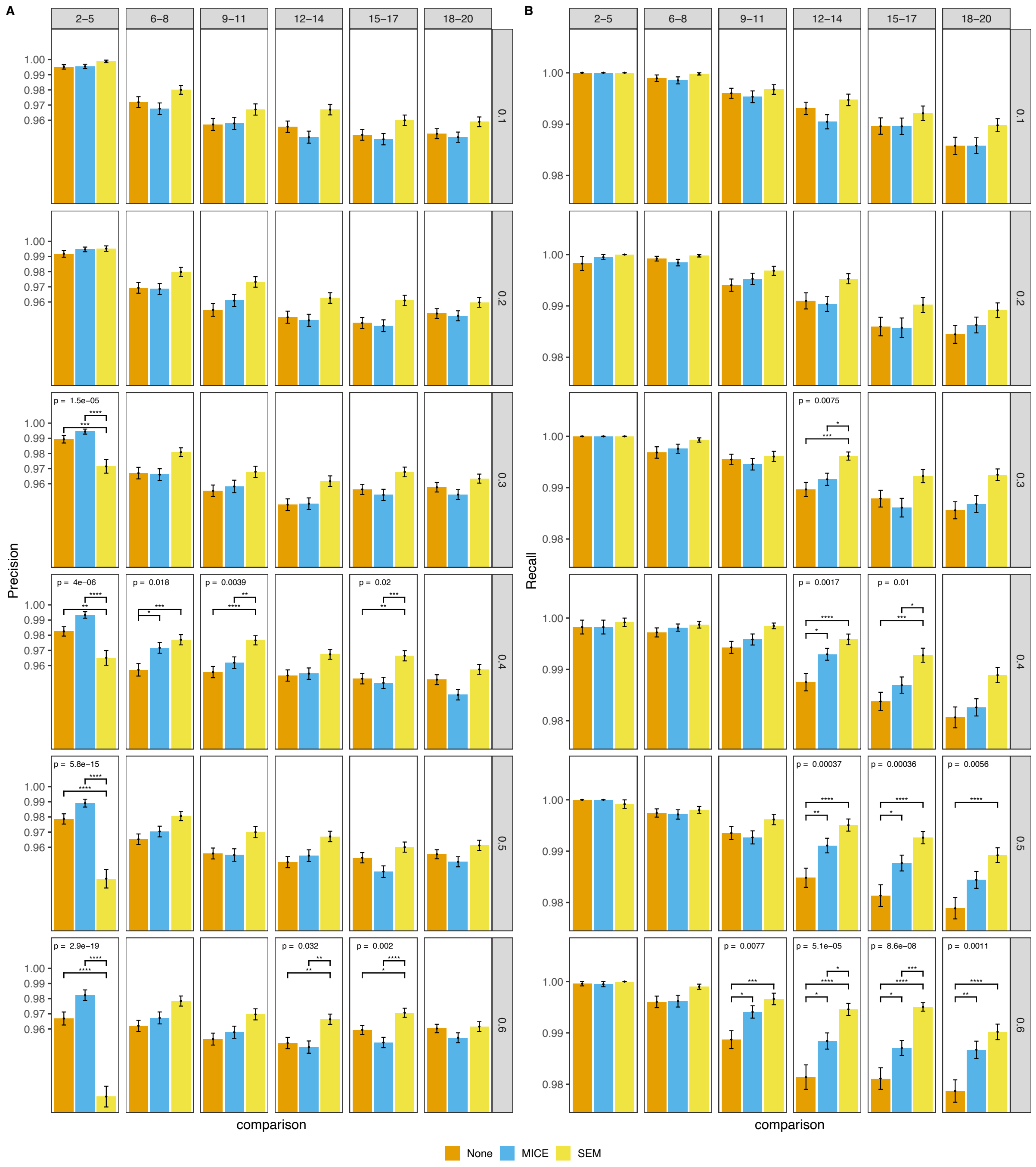

Supplementary Figure 6. Performance on MAR data with 10000 data points. Precision (A) and recall (B) of three different methods of handling incomplete data: none, multiple imputation by chained equations (MICE) and structural expectation-maximization (SEM). Rows represent different missing proportions and columns indicate different groups of number of variables. Barplots show means with error bars representing standard error of the mean. Adjusted p-values for ANOVAs are displayed in those panels that are significant at least the 0.05 level. Lines representing significant Tukey's HSD pairwise tests are shown and annotated as: *, $p < 0.05$; **, $p < 0.01$; ***, $p < 0.001$; ****, $p < 0.0001$.

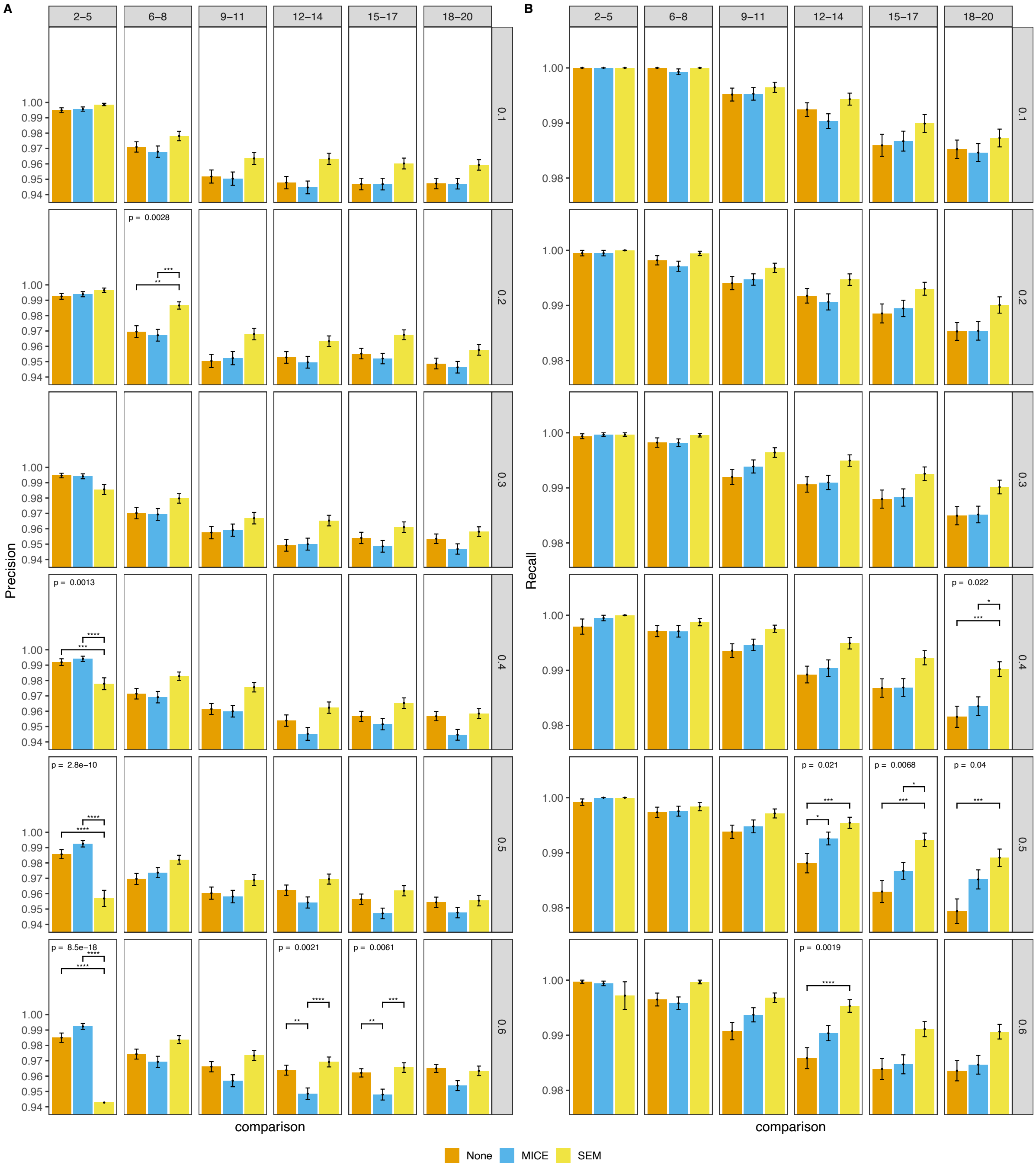

Supplementary Figure 7. Performance on MNAR data with 10000 data points. Precision (A) and recall (B) of three different methods of handling incomplete data: none, multiple imputation by chained equations (MICE) and structural expectation-maximization (SEM). Rows represent different missing proportions and columns indicate different groups of number of variables. Barplots show means with error bars representing standard error of the mean. Adjusted p-values for ANOVAs are displayed in those panels that are significant at least the 0.05 level. Lines representing significant Tukey's HSD pairwise tests are shown and annotated as: *, p < 0.05; **, p < 0.01; ***, p < 0.001; ****, p < 0.0001.

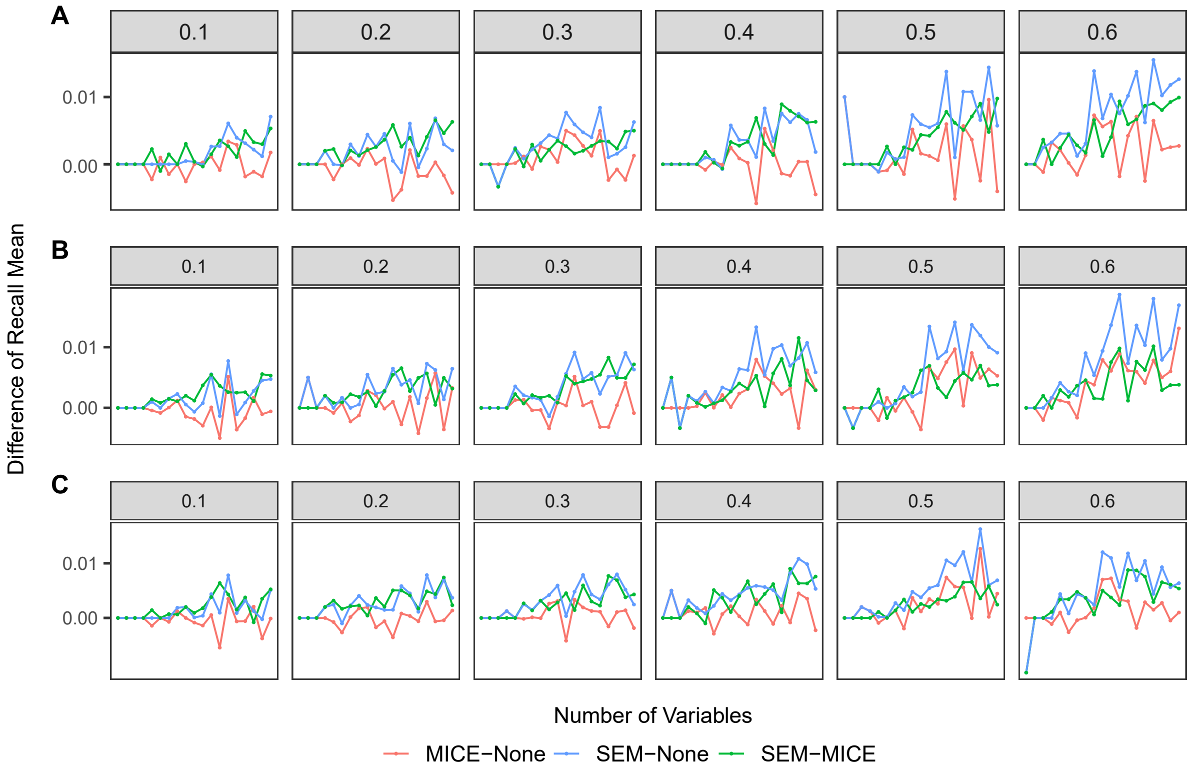

Supplementary Figure 8. Distribution of the difference in means of recall of three pairwise comparisons among three methods when there are 10000 data points: MICE's increase over using only complete cases (red), SEM's increase over nothing (blue), and SEM's increase over MICE (green) A. MCAR data. B. MAR data. C. MNAR data. The y-axis represents the difference of the mean recall (averaged over the 100 simulations). The x-axis represents the number of variables from 2-20. Column panels represent missing proportions.

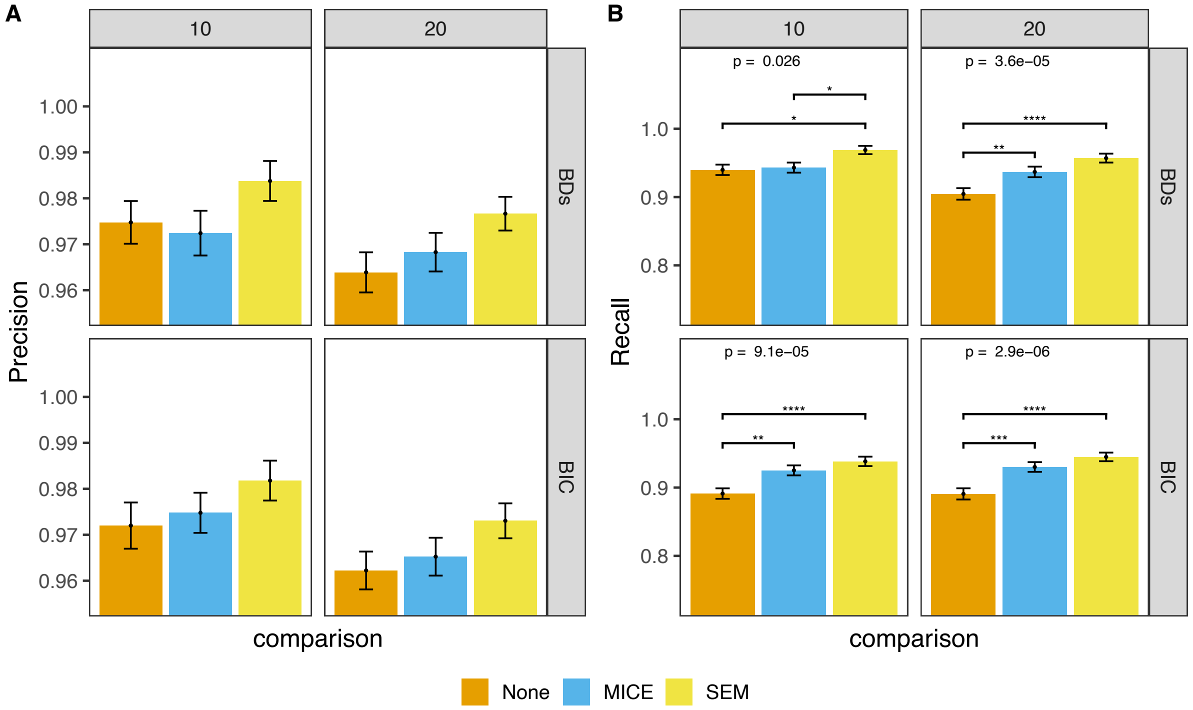

Supplementary Figure 9. Performance of BIC and BDs scores on MNAR data with 1000 data points and 0.3 missing proportion. Precision (A) and recall (B) of three different methods of handling incomplete data: none, multiple imputation by chained equations (MICE) and structural expectation-maximization (SEM). Columns represent different number of nodes (10 nodes and 20 nodes) and rows indicate different scoring matrices (BDs and BIC). Barplots show means with error bars representing standard error of the mean. Adjusted p-values for ANOVAs are displayed in those panels that are significant at least the 0.05 level. Lines representing significant Tukey's HSD pairwise tests are shown and annotated as: *, p < 0.05; **, p < 0.01; ***, p < 0.001; ****, p < 0.0001.

Supplementary Table 1. Description of variables in the real-world data set.

	Variables	Original Variables	Description	Levels
1	age	r13agey_e	age (years) of respondents at interview end month	"50 to 60"
				"60 to 70"
				"70 to 80"
				"above 80"
2	education	raeduc	education level of respondents	"less than high school"
				"high school graduate"
				"college and above"
3	race	raracem	race of respondents	"white/caucasian"
				"black/african american"
				"other"
4	gender	ragender	gender of respondents	"Male"
				"Female"
5	partnership	r13mstat	Partnership status of respondents	"currently married/ in partnership"
				"never married or partnered"
6	idincome	r13iearn r13ifearn	individual earnings of respondents (quantiles)	"0"
				"(0,2.5]"
				"(2.5,+04]"
7	hhincome	h13itot h13iftot	total household income of respondents (quantiles)	"[0,2.05e+04]"
				"(2.05e+04,4.3e+04]"
				"(4.3e+04,8.8e+04]"
				"(8.8e+04,1e+07]"
8	A1C	PA1CUW	HbA1c measure of respondents	"high risk (above or equal to 6.5%)"
				"low risk (below 6.5%)"
9	HDL	PHDLUW	HDL cholesterol measure of respondents	"high risk (below or equal to 40mg/dL)"
				"low risk (above 40mg/dL)"
10	tot_choles	PTCUW	total cholesterol measure of respondents	"high risk (above or equal to 240mg/dL)"

				"low risk (below 240mg/dL)"
11	BMI	r13bmi	obesity with body mass index (BMI) of respondents	"high risk (above or equal to 30kg/m2)"
				"low risk (below 30kg/m2)"
12	diabetes	r13diab<br>r13diabe<br>r13diabs<br>r13diabf	diabetes status of respondents	"has never had diabetes"
				"has/had diabetes"
13	hbp	r13hibp<br>r13hibpe<br>r13hibps<br>r13hibpf	high blood pressure status of respondents	"has never had high blood pressure"
				"has/had high blood pressure"
14	smoking	r13smoken<br>r13smokev	smoking behaviour of respondents	"never"
				"past smoker"
				"current smoker"
15	exercise	r13vgactx<br>r13mdactx<br>r13ltactx	exercise pattern of respondents	"frequently (more than once a week)"
				"occasionally (less than once a week)"
				"never"
16	drinking	r13drinkd	drinking behaviour of respondents	"frequently (more than three times a week)"
				"occasionally (less than or equal to three times a week)"
				"never"
17	cancer	r13cancr<br>r13cancre<br>r13cancrs<br>r13cancrf	cancer status of respondents	"has never had cancer"
				"has/had cancer"
18	lung	r13lung<br>r13lunge<br>r13lungs<br>r13lungf	lung disease status of respondents	"has never had lung disease"
				"has/had lung disease"

19	heart	r13heartr r13hearte r13hearts r13heartf	heart disease status of respondents	"has never had heart disease"
				"has/had heart disease"
20	stroke	r13strok r13stroke r13stroks r13strokf	stroke status of respondents	"has never had stroke"
				"has/had stroke"
21	arthritis	r13arthr r13arthre r13arthrs r13arthrf	arthritis status of respondents	"has never had arthritis"
				"has/had arthritis"
22	memory	r13pstmem	self-assessed memory decline	"better"
				"same"
				"worse"
23	TICS_M	r13ser7 r13imrc r13dlrc r13bwc20	cognitive impairment measure of respondents	"has cognitive impairment (total score below or equal to 11)"
				"no cognitive impairment (total score above 11)"
24	hbp_ treatment	r13rxhibp	whether respondents have ever taken treatment for high blood pressure	"Yes"
				"No"
25	diabetes_ treatment	r13rxdiabo r13rxdiabi r13rxdiab	whether respondents have ever taken treatment for diabetes	"Yes"
				"No"
26	heart_ treatment	r13rxheart	whether respondents had ever taken treatment for heart disease	"Yes"
				"No"
27	stroke_ treatment	r13rxstrok	whether respondents had ever taken treatment for stroke	"Yes"
				"No"
28	chol_ treatment	r13rxchol	whether respondents have ever taken treatment for cholesterol	"Yes"
				"No"
29	cancer_ treatment	r13cncrchem r13cncrsurg r13cncrradn r13cncrothr r13cncrmeds	whether respondents have ever taken treatment for cancer	"Yes"
				"No"

We used total score on a modified version of telephone interview for cognitive status measurement (TICS-M) to assess cognitive impairment (Hale, Schneider et al. 2020)

Variables r13rxstrok, r13rxheart, r13rxlung, r13cncrchem, r13cncrsurg, r13cncrradn, r13cncrothr, r13cncrmeds, r13rxhibp, r13rxdiabo, r13rxdiabi and r13rxdiab are from Harmonized HRS (VERSION C); variables PA1CUW, PHDLUW, and PTCUW are from 2016 Biomarker Data (Early, Version 1.0); the other variables are from RAND HRS Longitudinal File 2018 (V1).

Supplementary Table 2. Descriptive statistics of random network structures (mean ± SE of 100 repeats).

Number of Nodes	Edge Density	Clustering Coefficient	Diameter
2	0.5±0	-	1.0±0
3	0.395±0.008	0.37±0.049	1.29±0.046
4	0.342±0.007	0.461±0.035	1.58±0.054
5	0.301±0.006	0.43±0.026	2.15±0.05
6	0.282±0.005	0.475±0.019	2.31±0.046
7	0.26±0.004	0.417±0.015	2.63±0.06
8	0.24±0.003	0.409±0.012	2.76±0.057
9	0.218±0.002	0.36±0.011	2.98±0.047
10	0.209±0.002	0.339±0.01	3.22±0.058
11	0.195±0.002	0.322±0.009	3.4±0.059
12	0.184±0.001	0.313±0.008	3.57±0.074
13	0.17±0.001	0.285±0.008	3.77±0.076
14	0.165±0.001	0.275±0.006	3.83±0.075
15	0.154±0.001	0.264±0.006	4.08±0.08
16	0.146±0.001	0.243±0.006	4.37±0.073
17	0.141±0.001	0.238±0.005	4.35±0.074
18	0.135±0.001	0.232±0.005	4.37±0.072
19	0.129±0.001	0.218±0.005	4.92±0.082
20	0.121±0.001	0.194±0.004	4.82±0.08

Supplementary Table 3. Descriptive statistics of the degree sequence of random network structures (mean ± SE of 100 repeats); values show mean number of nodes having degree as per column for networks with number of nodes as per row.

nodes	in-degree sequence summary				out-degree sequence summary											
	0	1	2	3	0	1	2	3	4	5	6	7	8	9	10	11
2	1±0	1±0	-	-	1±0	1±0	-	-	-	-	-	-	-	-	-	-
3	1.2±0.04	1.3±0.07	0.6±0.1	-	1.2±0.04	1.3±0.07	0.5±0.05	-	-	-	-	-	-	-	-	-
4	1.3±0.05	1.5±0.08	0.9±0.07	0.2±0.04	1.4±0.06	1.4±0.08	0.9±0.07	0.3±0.05	-	-	-	-	-	-	-	-
5	1.3±0.05	1.9±0.1	1.2±0.09	0.6±0.07	1.4±0.05	1.8±0.1	1.2±0.1	0.5±0.05	*	-	-	-	-	-	-	-
6	1.4±0.06	1.8±0.09	1.6±0.09	1.1±0.08	1.6±0.06	1.7±0.1	1.7±0.1	0.8±0.07	0.2±0.04	*	-	-	-	-	-	-
7	1.4±0.06	1.9±0.1	1.9±0.11	1.7±0.1	1.6±0.06	2.2±0.11	1.6±0.1	1±0.08	0.4±0.05	0.2±0.04	*	-	-	-	-	-
8	1.6±0.07	1.9±0.11	2.1±0.12	2.4±0.11	1.9±0.07	2.1±0.12	1.9±0.12	1.3±0.1	0.6±0.06	0.3±0.05	*	-	-	-		-
9	1.5±0.06	2±0.12	2.7±0.14	2.8±0.12	2±0.08	2.3±0.11	2.2±0.12	1.5±0.1	0.7±0.08	0.3±0.05	*	-	-	-	-	-
10	1.5±0.06	2±0.11	2.8±0.15	3.7±0.13	2.1±0.08	2.6±0.11	2.2±0.11	1.6±0.11	1±0.09	0.5±0.06	*	*	-	-	-	-
11	1.5±0.0	2±0.1	3.1±0.14	4.4±0.13	2.1±0.08	2.9±0.12	2.4±0.1	1.6±0.1	1.1±0.09	0.6±0.06	0.2±0.04	*	-	-	-	-
12	1.5±0.07	2±0.11	3±0.14	5.4±0.13	2.2±0.09	3.2±0.1	2.4±0.14	2±0.12	1.3±0.1	0.7±0.07	0.2±0.04	*	*	-	-	-
13	1.5±0.0	2.2±0.12	3.7±0.17	5.7±0.16	2.5±0.11	3.4±0.15	2.6±0.14	1.9±0.11	1.4±0.1	0.7±0.08	0.4±0.05	*	*	*	-	-
14	1.4±0.05	2.1±0.1	3.8±0.17	6.8±0.16	2.7±0.1	3.4±0.14	2.8±0.14	2.2±0.12	1.6±0.12	0.8±0.08	0.4±0.06	0.2±0.04	*	*	-	-
15	1.5±0.05	2.2±0.11	3.8±0.15	7.5±0.15	2.8±0.1	3.5±0.16	3.1±0.17	2.5±0.14	1.5±0.11	0.9±0.08	0.5±0.06	0.2±0.04	*	*	-	-
16	1.5±0.06	2.2±0.12	4±0.17	8.4±0.16	2.9±0.11	3.6±0.2	3.5±0.16	2.6±0.15	1.7±0.12	1.1±0.09	0.5±0.07	0.2±0.04	*	*	*	-
17	1.4±0.06	2.3±0.12	3.9±0.18	9.4±0.17	3±0.2	4±0.16	3.4±0.16	2.7±0.16	1.9±0.14	1.1±0.1	0.7±0.08	0.2±0.05	*	*	-	-
18	1.4±0.06	2.09±0.11	4.2±0.17	10.2±0.17	3.1±0.11	4.3±0.15	3.6±0.15	2.8±0.13	1.9±0.11	1.2±0.09	0.6±0.07	0.4±0.05	0.1±0.03	*	*	-
19	1.5±0.06	2.35±0.104	4±0.16	11.2±0.17	3.1±0.1	4.6±0.17	3.9±0.15	3±0.15	1.8±0.12	1.2±0.09	0.8±0.07	0.4±0.06	0.2±0.05	*	-	*
20	1.5±0.06	2.5±0.1	4.4±0.17	11.7±0.15	3.5±0.1	4.6±0.16	4±0.16	3.2±0.13	2.3±0.14	1.3±0.10	0.7±0.07	0.3±0.05	0.1±0.04	*	*	-

* indicates there are less than 10 networks having 1 node of the specified degree value.

References

Hale, J. M., D. C. Schneider, J. Gampe, N. K. Mehta and M. Myrskyla (2020). "Trends in the Risk of Cognitive Impairment in the United States, 1996-2014." Epidemiology **31**(5): 745-754.
